# Sesamol Induces Human Hepatocellular Carcinoma Cells Apoptosis by Impairing Mitochondrial Function and Suppressing Autophagy

**DOI:** 10.1038/srep45728

**Published:** 2017-04-04

**Authors:** Zhigang Liu, Bo Ren, Yihui Wang, Chen Zou, Qinglian Qiao, Zhijun Diao, Yashi Mi, Di Zhu, Xuebo Liu

**Affiliations:** 1 Laboratory of Functional Chemistry and Nutrition of Food, College of Food Science and Engineering, Northwest A&F University, Yangling, Shaanxi 712100, China; 2 School of Medicine, Tulane University, New Orleans, LA 70112, USA; 3 School of Agriculture and Biology, Shanghai Jiao Tong University, Shanghai 200240, China

## Abstract

Sesamol, a nutritional phenolic antioxidant compound enriched in sesame seeds, has been shown to have potential anticancer activities. This study aims at characterizing the antitumor efficacy of sesamol and unveiling the importance of mitochondria in sesamol-induced effects using a human hepatocellular carcinoma cell line, HepG2 cells. Results of this study showed that sesamol treatment suppressed colony formation, elicited S phase arrest during cell cycle progression, and induced both intrinsic and extrinsic apoptotic pathway *in vitro* with a dose-dependent manner. Furthermore, sesamol treatment elicited mitochondrial dysfunction by inducing a loss of mitochondrial membrane potential. Impaired mitochondria and accumulated H_2_O_2_ production resulted in disturbance of redox-sensitive signaling including Akt and MAPKs pathways. Mitochondrial biogenesis was inhibited as suggested by the decline in expression of mitochondrial complex I subunit ND1, and the upstream AMPK/PGC1α signals. Importantly, sesamol inhibited mitophagy and autophagy through impeding the PI3K Class III/Belin-1 pathway. Autophagy stimulator rapamycin reversed sesamol-induced apoptosis and mitochondrial respiration disorders. Moreover, it was also shown that sesamol has potent anti-hepatoma activity in a xenograft nude mice model. These data suggest that mitochondria play an essential role in sesamol-induced HepG2 cells death, and further research targeting mitochondria will provide more chemotherapeutic opportunities.

Mitochondria are the main cellular energy sources that generate ATP through the process of respiration and oxidative phosphorylation (OXPHOS) under normal physiological and pathological conditions^1^. Unlike normal cells, many cancer cells derive a substantial amount of energy from aerobic glycolysis, converting most incoming glucose to lactate rather than through OXPHOS in the mitochondria. However, mitochondria still play a central and multifunctional role in the proliferation and growth of these malignant tumor cells, which indicates the therapeutic potential in targeting mitochondria^2^,^3^,^4^. It has been shown that excess reactive oxygen species (ROS) produced by mitochondria lead to cell death^5^. The BCL-2 family of proteins at the mitochondrial outer membrane mediate apoptosis by controlling the release of cytochrome *c* from the mitochondrial intermembrane space, which triggers the caspase protease activation in cytosol^6^. Cellular survival- and death- signals such as 3-kinase/protein kinase B (PI3K/Akt) and mitogen-activated protein kinases (MAPKs) are also regulated by mitochondrial signaling^7^.

Autophagy enables tumor cell survival by enhancing stress tolerance. This enhanced stress tolerance is exhibited through recycling cellular components and metabolic regulation thus reducing damage and sustaining viability^8^. It is a highly conserved and genetically programmed process for removing aggregated proteins and unwanted organelles, including damaged mitochondria. Mitochondrial autophagy, or mitophagy, is a major mechanism involved in mitochondrial quality control via selectively degrading damaged or unwanted mitochondria. Recent studies demonstrated that mitophagy also plays a pivotal role in regulating cancer cell death^9^. Insufficient mitophagy process impairs recycling and results in accumulation of dysfunctional mitochondria, which may contribute in malignant transformation^10^. Furthermore, autophagy plays an essential role in supporting rapid tumor cell proliferation and maintaining tumor cell metabolic function via lysosomal-mediated degradation^11^. Several rodent models indicate that inhibition of autophagy leads to the impairment of mitochondrial metabolism and a deficiency in ATP production *in vivo*. Accumulation of abnormal mitochondria was also found in autophagy-deficient tumors, which resulted in the loss of cellular metabolic homeostasis^12^,^13^.

Sesamol, a liposoluble lignans extraction and prominent fragrance component in sesame oil, has been demonstrated to exhibit potential anticancer activities. It has been exclusively reported to induce apoptosis in different cancer cells including rodent leydig tumor cells, gastric cancer cells, skin cancer cells, and human colon cancer cells^14^,^15^,^16^,^17^. Our previous research also implicated that sesamol efficiently induces the apoptosis of human liver hepatocellular carcinoma (HepG2) cells^18^. Although recent reports indicated that sesamol treatment induced mitochondrial apoptosis in skin cancer and colon cancer cell line^19^,^20^, the mechanism of sesamol-induced HepG2 cells apoptosis is still unclear.

The purpose of our current study is to (a) characterize the role of sesamol on HepG2 cell’s mitochondrial membrane potential and whole cell energy metabolism, a potential process that has been overlooked; (b) investigate the link between sesamol-mediated mitochondrial dysfunction and modulation of survival- and death-signaling, and (c) determine the effects and mechanisms of sesamol on mitophagy and autophagy, and their impacts on apoptosis.

## Results

### Sesamol suppressed cell colonization and induced cell cycle arrest and apoptosis in HepG2 cells

**Figure 1:**
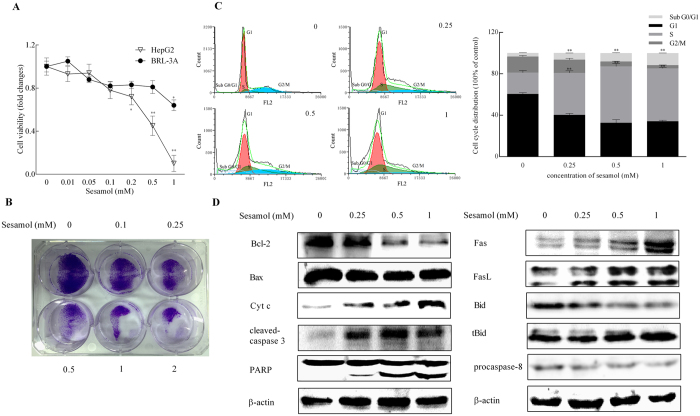
Sesamol inhibited cell viability, induced cell cycle arrest, and stimulated intrinsic and extrinsic apoptosis in HepG2 cells. Cells were treated with sesamol at the indicated concentrations for 24 h. (**A**) Viability changes of HepG2 cells and BRL-3A cells after various concentration of sesamol treatment; (**B**) Sesamol potently inhibited colony formation of HepG2 cells. Cells were seeded into 6-well plates. After incubation for 24 h, cells were subjected to treatments with serial concentrations of sesamol. After 8 days of incubation, the numbers of colony formation were photographed in the methods. (**C**) Flow cytometric analysis to detect cell cycle. After sesamol treatment, HepG2 cells were harvested and the distribution of cell cycle was exhibited by flow cytometric analysis. (**D**) Representative western blots of expressions of intrinsic apoptotic proteins: Bcl-2, Bax, cytochrome *c*, cleaved-caspase-3, PARP, and extrinsic apoptotic proteins: Fas, FasL, Bid, tBid, procaspase 8. Data presented as mean ± SD, n ≥ 6 wells per group, *p < 0.05, **p < 0.01 versus blank treatment group.

Previous studies indicated that sesamol reduced cell viability and induced apoptosis in HepG2 cells^18^. We have shown here that sesamol significantly impaired HepG2 cells viability in a dose-dependent manner with much lower cytotoxicity on normal hepatocytes BRL-3A cells (Fig. 1A). To further evaluate the effects of sesamol on cancer cell growth, a colony formation assay was conducted on HepG2 cells (Fig. 1B). Compared to untreated group, sesamol treatment showed potent and concentration-dependent inhibitory effects on the colony formation of HepG2 cells after treated for 24 h. Moreover, the ratio of Sub G0/G1 was increased 8%, the ratio of G1 phase cells decreased 16%, while the S phase cells increased 31% after sesamol treatment with the dose range from 0 to 1 mM (Fig. 1C), which indicated that sesamol treatment triggered apoptosis and cell cycle arrest at S phase in HepG2 cell.

To determine the apoptotic pattern induced by sesamol, we examined both intrinsic and extrinsic apoptosis pathways. For intrinsic apoptosis pathway, sesamol decreased the expression of anti-apoptotic protein Bcl-2, but showed no effect on the expression of apoptotic signal Bax. Meanwhile, sesamol improved the release of cytochrome *c* from mitochondria, which further increased the cleavage of caspase-3 (the initiator- and effector caspases in the intrinsic apoptotic pathway) as well as poly-ADP-ribose polymerase (PARP) (Fig. 1D). Simultaneously, sesamol improved the protein expression of Fas/FasL, and activated tBid and caspase-8 which are all involved in the extrinsic apoptosis pathway. These data suggested that sesamol suppressed cell proliferation and induced intrinsic and extrinsic apoptosis in HepG2 cells.

### Sesamol elicited mitochondrial dysfunction, cellular redox status imbalance and redox-sensitive signaling disruption in HepG2 cells

**Figure 2:**
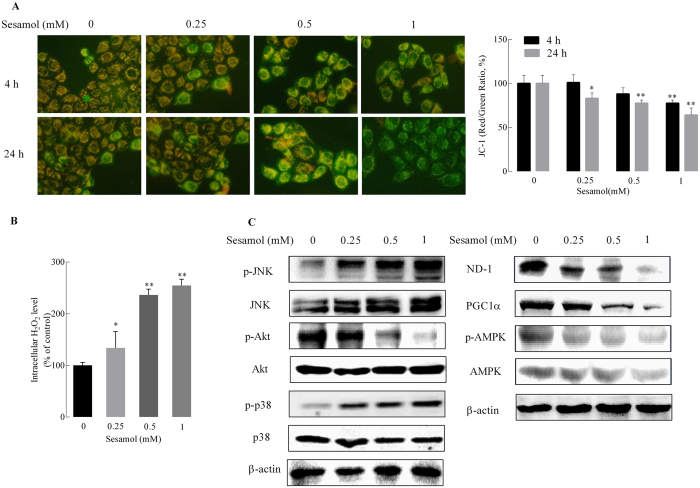
Effects of sesamol on mitochondrial membrane potential and redox-sensitive signaling in HepG2 cells. Cells were treated with sesamol at the indicated concentrations for 4 or 24 h. After treatment, (**A**) the cells were detected by a multimode reader after staining with 5 μg/mL JC-1, and were photographed by fluorescence microscopy; the bar graph is the fluorescence intensity which was measured using a multimode microplate reader at 485 nm excitation, 585 nm (red/orange for normal MMP) and 538 nm (green for loss of MMP) emission, respectively. (200 × , magnification). (**B**) H_2_O_2_ production was determined by the Amplex Red Hydrogen Peroxide/Peroxidase Assay, and (**C**) Left panel is the representative western blots of expressions of redox-sensitive signaling Akt, and MAPK signaling JNK and p38; and the right panel is the representative western blots of expressions of mitochondria complex I subunit ND1 and mitochondrial biogenesis related protein PGC1α, pAMPK/AMPK. Data presented as mean ± SD, n ≥ 6 wells per group, *p < 0.05, **p < 0.01 versus control group.

Mitochondrial membrane potential (MMP) is an important indicator of mitochondrial function. MMP loss is also a characteristic of cell apoptosis^21^. HepG2 cells treated with sesamol showed a substantial decrease in MMP in a concentration- and time-dependent manner. Compared to the control group, sesamol significantly caused the loss of MMP by 22.5% at the highest concentration (1 mM) for 4 h treatment. After 24 h, sesamol induced MMP loss at all concentrations tested from as low as 0.25 mM; and MMP decreased by 36.1% at the highest concentration (1 mM) (Fig. 2A). However, the same concentration of sesamol showed no effects on MMP of BRL-3A cells (see Supplementary Fig. S1A).

The cellular redox status partially depends on the production of H_2_O_2_, which has been considered as a second messenger in the redox regulation of cell signaling and transcription. Mitochondria play pivotal roles in regulating cellular redox status by release of H_2_O_2_, and mediating redox-sensitive signaling pathway, such as mitogen-activated protein kinases MAPKs and PI3K/Akt pathways^22^. As shown in Fig. 2B, sesamol substantially stimulated H_2_O_2_ production in a concentration-dependent manner, which is consistent with the MMP loss induced by sesamol. Alternatively, the same concentration of sesamol did not lead to an oxidized status in BRL-3A cells (see Supplementary Fig. S1B).

PI3K/Akt is involved in the regulation of cell survival via the maintenance of the bioenergetic and metabolic capacities of mitochondria. Conversely, MAPK kinases, JNK and p38, activate apoptotic signaling by either upregulating the expressions of pro-apoptotic genes via transactivation of specific transcription factors or directly modulating the activities of pro- and anti-apoptotic proteins through distinct phosphorylation events. Here we showed that sesamol activated Akt and inactivated the JNK/p38 pathway at various concentrations (Fig. 2C). Interestingly, sesamol also decreased the protein expressions of mitochondrial complex I subunit ND1 and mitochondrial biogenesis-related signal protein peroxisome proliferator-activated receptor gamma coactivator 1-α (PGC1α). The phosphorylation of Adenosine monophosphate activated protein kinase (AMPK) was also suppressed by sesamol in a dose-dependent manner. Overall, sesamol triggered mitochondrial dysfunction and excess H_2_O_2_ production in HepG2 cells, which consequently unbalanced redox-sensitive signaling through Akt and MAPKs pathways.

### Sesamol suppressed autophagy and mitophagy in HepG2 cells

**Figure 3:**
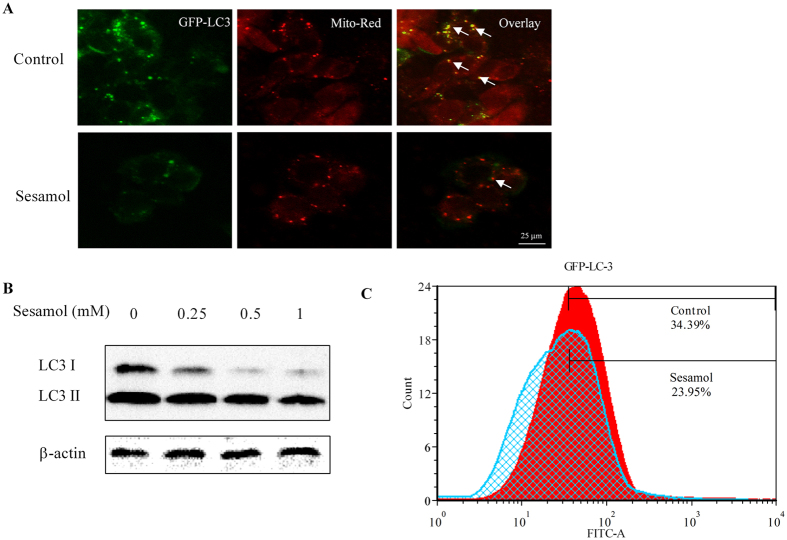
Sesamol inhibited mitophagy and autophagy in HepG2 cells. (**A**) HepG2 cells were transfected with GFP-LC3 virus for 16 h, then pre-treated with/without 100 nM rapamycin for 30 min, followed by treatment with 1 mM sesamol for 24 h. Green channel demonstrated GFP-LC3. Red channel demonstrated mitochondria by MitoTracker-red staining. In the overlay channel, the mitophagy was shown as orange. (**B**) HepG2 cells were treated with sesamol at the indicated concentrations for 24 h. Representative western blots of expressions of LC3 I and LC3 II. (**C**) Flow cytometry analysis of GFP-LC3 after HepG2 cells were transfected with GFP-LC3 for 16 h and treated with 1 mM sesamol for 24 h.

Autophagy and mitophagy play essential roles in regulating tumor cell survival and death^23^. Light Chain 3 (LC3), an indicator of autophagy, is associated with autophagic vesicles formation. As shown in Fig. 3A, the co-localizing of GFP- labelled LC3 and mitochondria (stained by Mito-Red fluorescent agent) was decreased after sesamol treatment, which indicated that sesamol blocked the mitophagy process in HepG2 cells. After sesamol treatment for 24 h (without serum media), the expression of LC3 I and LC3 II enormously decreased compared to control group (Fig. 3B). This decrease in LC3 expression confirmed using flow cytometry as shown in Fig. 3C. These data indicated that sesamol suppressed autophagy in HepG2 cells.

### Sesamol induced apoptosis through suppression of autophagy in HepG2 cells

**Figure 4:**
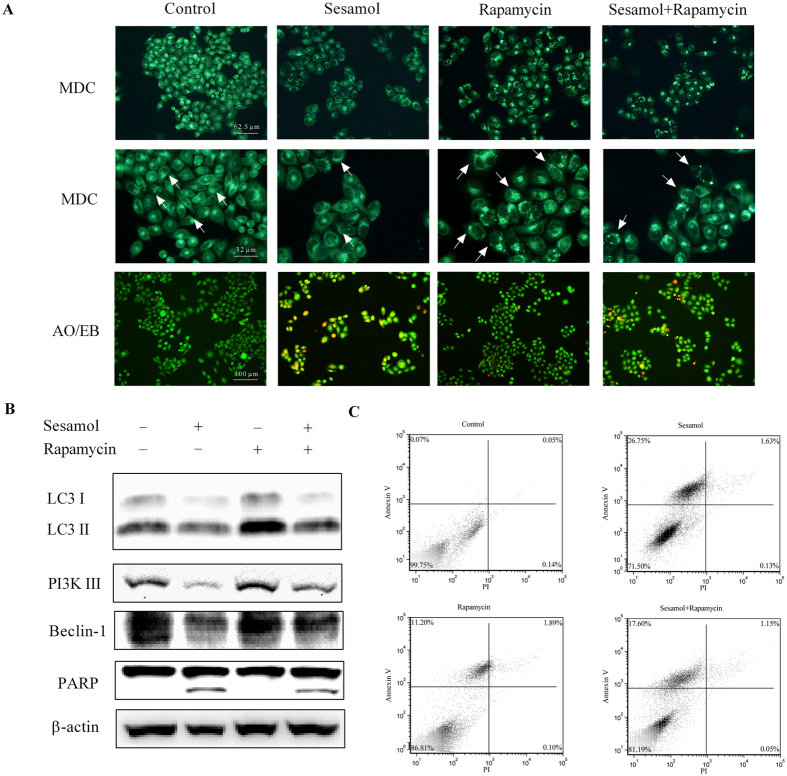
Stimulation of autophagy by rapamycin ameliorated sesamol-induced apoptosis of HepG2 cells. HepG2 cells were pre-treated with/without 100 nM rapamycin for 30 min, and then treated with 1 mM sesamol for 24 h. (**A**) The effect of autophagy stimulator rapamycin on sesamol-induced HepG2 cells. Autophagy was detected by MDC staining and apoptosis was examined by AO/EB staining, as described in Materials and Methods section. (**B**) Representative western blots of expressions of LC3 I, LC3 II, PI3K III, Beclin-1, and PARP after sesamol treatment with/without 100 nM rapamycin pre-treatment. (**C**) Cells were stained with annexin-V (AV) and propidium iodide (PI) to distinguish between unaffected cells (Annexin V negative, PI negative), and apoptotic cells (Annexin V positive, PI negative by flow cytometry).

Rapamycin is widely reported as an autophagy stimulator^24^. Monodansylcadaverine (MDC) works as a stain in labeling cellular vesicles^25^. Our data suggested sesamol significantly decreased the vesicles numbers, even in the cells that were co-treated with rapamycin in HepG2 cells (Fig. 4A). Acridine orange (AO)/ethidium bromide (EB) staining was employed as indicator for cell apoptosis^18^. Rapamycin partially prevented sesamol induced apoptosis in HepG2 cells (Fig. 4A). To better investigate how sesamol suppressed autophagy, PI3K Class III/Beclin-1 signaling, an important autophagy-regulating pathway^26^, was examined after sesamol treatment with/without the addition of rapamycin. Results in Fig. 4B indicated that sesamol inhibited the expression of LC3 through suppressing the expressions of PI3K Class III and Beclin-1.

Moreover, to investigate the involvement of impaired autophagy in sesamol-induced apoptosis, we examined Annexin V/PI labeled HepG2 cells by flow cytometer. Fig. 4C demonstrated that autophagy activator, rapamycin, reduced sesamol-induced apoptosis from 26.75% to 17.6%, which is consistent with rapamycin partially reversed cleavage of PARP after sesamol treatment and AO/EB staining results. These data suggested that sesamol elicited apoptosis through inhibition of autophagy.

### Sesamol elicited energy metabolism disruption in HepG2 cells

**Figure 5:**
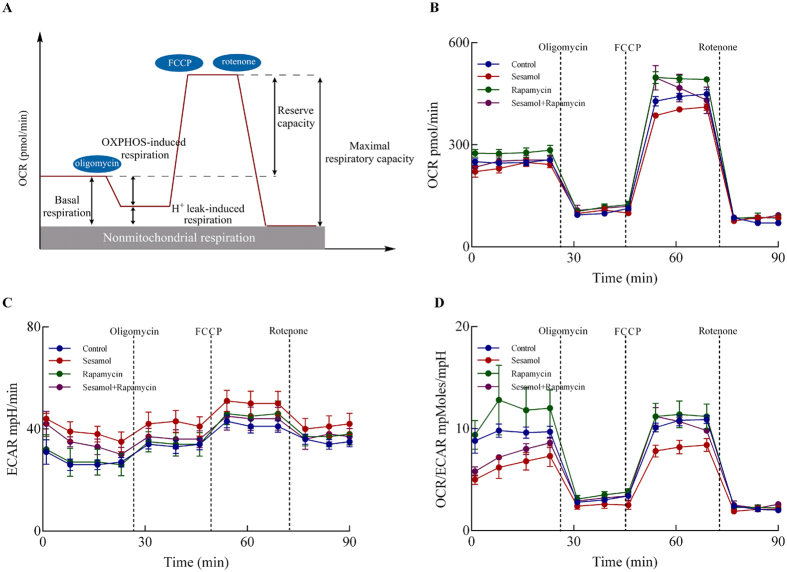
Effects of sesamol on metabolism in HepG2 cells. (**A**) Illustration of bioenergetic parameters from energy flux analysis. (**B**) Time course of oxygen consumption rate (OCR), (**C**) the extracellular acidification rate (ECAR), and (**D**) the ratio of OCR and ECAR determined using XF-24 Extraflux analyzer as described under Materials and Methods. Data presented as mean ± SD, n = 5 wells per group, *p < 0.05, **p < 0.01 versus control group.

To evaluate the effects of sesamol on energy metabolism in HepG2 cells, especially on aerobic glycolysis and OXPHOS in the mitochondria, the metabolic flux analysis was performed as described in Methods & Materials section. The oxygen consumption rates (OCR) parameters indicate the mitochondrial energy metabolism in HepG2 cells (Fig. 5A): the decrease in basal respiration after addition of oligomycin served as an indicator for ATP production and the further addition of FCCP to uncouple mitochondrial respiration from oxidative phosphorylation yielded the maximal respiration. After treated with mitochondrial respiration inhibitor, rotenone, the OCR value of HepG2 cells declines. The difference between maximal respiration rate and basal respiration is indicated as the spare respiratory capacity. The basal respiration, OXPHOS-induced respiration, H^+^ leak-induced respiration, reserve capacity, and maximal respiratory capacity were calculated as indicated in Fig. 5A. Besides, ECAR values indicate the lactate formation in HepG2 cells. Sesamol treatment significantly impacted the mitochondrial respiration and anaerobic glycolysis, as indicated by OCR (Fig. 5B) and extracellular acidification rates (ECAR) (Fig. 5C). Sesamol treatment decreased the basal respiration and maximal respiratory capacity by 13.1% and 13.2%, respectively, but had no effect on OXPHOS-induced respiration, H^+^ leak, nonmitochondrial respiration, and reserve capacity compared to control group (Table 1). Interestingly, sesamol elevated the ECAR curve. Autophagy stimulator rapamycin partially restored sesamol impaired mitochondrial respiration with improved maximal respiratory capacity by 19.2% (Table 1). The ratio of OCR/ECAR was also increased by rapamycin treatment compared to the sesamol treatment group. These data indicated that sesamol treatment suppressed mitochondrial metabolism in HepG2 cells, which might be associated with its inhibitory effects on autophagy.

### Sesamol inhibited tumor growth and induced cell apoptosis *in vivo*

**Figure 6:**
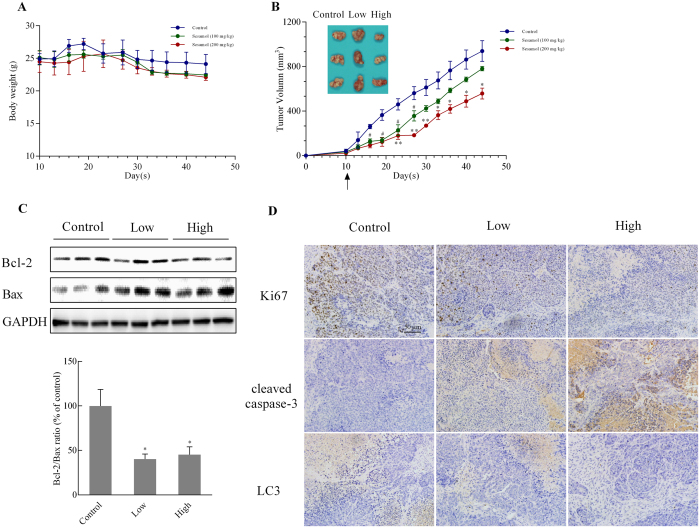
Sesamol suppressed tumor growth in nude mice. To generate xenograft tumor model, the nude mice (Balb/c nu/nu) were injected subcutaneously with 1 × 10^7^/mL HepG2 cells, and then treated with sesamol (100 mg/kg or 200 mg/kg) or saline. (**A**) The body weight changes; (**B**) Representative pictures of tumors in each group, and the xenograft tumors volume curve of each group. (**C**) The protein expressions of Bcl-2, Bax, and their ratio after sesamol treatment in tumor tissue. (**D**) The expression of Ki67, cleaved-caspase 3, and LC3 was analyzed using IHC. Data presented as mean ± SEM, n = 5 mice per group, ^#^p < 0.05, ^##^p < 0.01, low dose sesamol treatment group versus control group, *p < 0.05, **p < 0.01 high dose sesamol treatment group versus control group.

To evaluate the antitumor activities of sesamol *in vivo*, we established a human hepatocellular carcinoma cells (HCC) xenograft model by subcutaneously injecting HepG2 cells into the backs of nude mice. Sesamol treatment for 35 days did not significantly impact body weight (Fig. 6A), although the tumor volume was dramatically inhibited (40.56% size inhibitory rate with sesamol at 200 mg/kg compared to the control group) (Fig. 6B). However, a lower dose (100 mg/kg sesamol) only had significant anti-tumor growth effect up to 27 days after the first treatment. The Bcl-2/Bax ratio in tumor tissues was also decreased after sesamol treatment (Fig. 6C), which is consistent with our *in vitro* study. Moreover, in the sesamol treatment group, levels of the cell proliferation marker Ki76 were down-regulated, and levels of the cell apoptosis marker cleaved-caspase 3 were increased when compared to control (Fig. 6D). The expression of LC3 protein was remarkably decreased by sesamol in a dose-dependent manner.

## Discussion

As reported, sesamol is an efficient antioxidant with potential therapeutic benefits in various tumor cells and animal models. Sesamol exhibits a radioprotective effect by preventing DNA damage and enhancing DNA repair afterγ-radiation^27^,^28^,^29^. It was also reported that sesamol, similar with resveratrol, had almost equally potent chemopreventive effect on skin cancer in a mouse model^16^. In addition, previous studies indicated that sesamol had hepatoprotective effects in cyclophosphamide, carbon tetrachloride, acetaminophen, lead, or nutrition deficiency induced liver damage^30^,^31^,^32^,^33^,^34^. Our lab has shown sesamol possessed cytotoxicity in hepatocellular carcinoma cells in a previous publication^18^. There have been numerous of reports in the literature that sesamol possesses curative role in various diseases due to its diverse bioactivities *in vivo* such as anti-inflammatory, lipid-lowering, neuroprotective, and chemopreventive^19^,^35^,^36^,^37^. Previous study have demonstrated that oral administration of sesamol was absorbed and metabolized in the liver, which are then excreted in the bile or urine. A recent biopharmaceutical profiling study also proved that sesamol has relative high oral bioavailability (95.61%). Sesamol and its conjugated metabolites were widely distributed in tissues such as kidneys, lungs, brain, and liver^38^. The pKa of sesamol was 9.79 and distribution coefficient of >1, with a quite small the t_1/2_ value, which indicated that the elimination of sesamol is fast^39^,^40^. However, high dose (>50 mg/kg) of sesamol was also reported to cause hyperactivity in rats^41^. Besides, adequate studies suggested that sesamol induced cancer cell apoptosis at a dose range from 0.1 to 1 mM, yet there is one report indicated that a higher dose (>0.25 mM) of sesamol may induce blood platelets damage^42^. This potentially toxic effect highlights the critical need for proper dosage when sesamol is developed for clinical use.

Our data has demonstrated that sesamol inhibited colony formation of HepG2 cells, which is consistent with the previous result that sesamol impaired cancer cell viability. We have also observed the accumulation of sesamol in nucleus^18^. Our previous data indicated that sesamol could interact with DNA at its minor groove. In this study, we showed sesamol induced cell cycle arrest at S phase in a concentration-dependent manner (Fig. 1), which suggested that sesamol induced DNA damage or blocked DNA replication. The increase ratio of sub G0/G1 indicated that sesamol treatment trigger apoptosis in HepG2 cells. Furthermore, our hepatoma xenograft model indicated treatment with sesamol inhibited proliferation and induced apoptosis of tumor cells *in vivo* (Fig. 6). However, the optimized dose of sesamol is still under investigation for cancer chemotherapeutic use *in vivo* due to its potential cytotoxicity in normal cells.

Phytochemicals, such as quercetin and resveratrol, from dietary plants and other plant sources are becoming increasingly important sources of compounds for cancer chemoprevention. Several candidates have been proven to inhibit cancer progression and metastasis after the formation of pre-neoplastic cells by interfering with cell cycle regulation, signal transduction, transcriptional regulation, and apoptosis^43^,^44^,^45^. For instance, curcumin and quercetin were reported recently to induce cancer cells or animal tumor cells apoptosis by activating both intrinsic and extrinsic pathway^46^,^47^. As mentioned before, mitochondria play an essential role in the intrinsic apoptotic pathway. Loss of MMP indicated the involvement of the intrinsic pathway in sesamol induced HepG2 cells apoptosis. However, mitochondria are also associated with the extrinsic pathway (i.e. death receptor pathway) through a pro-apoptotic “BH3 domain-only” member of the Bcl-2 family-Bid. Bid is normally localized in the cytosol as an inactive precursor and is cleaved during Fas signaling, leading to the translocation of the carboxyl terminal p15 fragment (tBid) to the mitochondrial outer membrane. This process is associated with the release of cytochrome *c* from the mitochondria, resulting in apoptosis^48^. Thus, Bid relays an apoptotic signal from the cell surface (sesamol activated Fas/FasL) to the mitochondria and triggers caspase activation (sesamol induced apoptosis).

Mitochondria are one of the main sources of radical oxygen species, such as H_2_O_2_. Most cancer cells require a higher level of oxidative stress than normal cells to maintain a balance between undergoing either proliferation or apoptosis^49^. The tolerable amounts of H_2_O_2_ function as signaling molecules in the MAPKs pathway to constantly activate redox-sensitive transcription factors such as NFκB and responsive genes that are involved in the survival of cancer. However, excess amounts of H_2_O_2_ cause cell cycle arrest or apoptosis^49^. Sesamol induced mitochondrial dysfunction and improved H_2_O_2_ production, thus increased oxidative stress in HepG2 cells accompanied by inactivation of Akt and stimulation of MAPKs signaling. As mentioned above, PI3K/Akt pathway is activated in most cancer cells. Akt is involved in the maintenance of the bioenergetic and metabolic capacities of mitochondria. Decreased phosphorylation of Akt by sesamol was consistent with the inhibited mitochondrial respiration.

Previous studies suggested that mitochondrial quality control is highly regulated by mitochondrial biogenesis and mitophagy^50^. Mitochondrial biogenesis is triggered by the general energy sensor AMP kinase and other mechanisms^51^. In this study, sesamol damaged mitochondrial energy production, and inhibited the phosphorylation of AMPK at the same time, which was consistent with the suppression of PGC1α, a controller and indicator for mitochondrial biogenesis (Fig. 2). To prevent cancer cell rapid growth and proliferation, we impaired the energy production and blocked the structural resource of HepG2 cells by treated them with serum-free medium for 24 h. With the depletion of an external nutrient, we were able to prove that autophagy plays an essential role in maintaining cellular metabolism via recycling of cellular components^52^. Under certain conditions autophagy can mitigate tumorigenesis by clearing damaged proteins and organelles, indicating some nonspecific means for stimulating autophagy such as caloric restriction and fasting may improve human health and suppress cancer^53^. However, autophagy can also be a survival process for tumor cells to tolerate metabolic and hypoxia stress. It is reported that an increased production of ribosomes was observed in cancer cells, which alters synthesis of specific proteins that control cell cycle progression and promote cell proliferation^54^. Under these circumstances autophagy inhibitors are expected for therapeutic use^55^. Sesamol inhibited autophagy by suppressing the PI3K Class III/Beclin-1 pathway, and further blocking LC3 expression and autophagosome formation (Fig. 3). Mitophagy inhibition by sesamol might improve accumulation of defective mitochondria, leading to a reduction of mitochondrial metabolism by blocking mitochondrial quality control and substrate supply. Likely, damaged or aged mitochondria generate more ROS to induce cancer cell death, which agreed with rapamycin reversed sesamol-induced apoptosis (Fig. 4). However, the underlying mechanism of inhibitory effects of sesamol on autophagy and how it influenced cancer cell survival aside from the dysregulation of mitochondrial quality control are still unclear.

Decades ago, Otto Warburg indicated that cancer cells ferment glucose in the presence of oxygen, suggesting their deficiency in mitochondrial respiration. Recent studies demonstrated however, that mitochondria still play essential roles in supplying energy, providing building blocks for cell proliferation, and controlling redox homeostasis and apoptosis in tumor cells^2^. Sesamol, in this research, suppressed mitochondrial complex I (Fig. 2) and blocked mitochondrial energy metabolism in HepG2 cells (Fig. 5), thus limiting the supply of energy for cell growth and proliferation. Conversely, the cellular anaerobic metabolism was improved after sesamol treatment, which might be a counterbalance for the loss of aerobic respiration. Consistently, various concentrations (0–1 mM) of sesamol treatment showed no significant change on HepG2 cells glucose consumption (see Supplementary Fig. S2). Rapamycin elevated autophagosome generation and improved mitochondrial metabolism^56^. As shown in Fig. 5B and D,rapamycin reversed sesamol impaired OCR and the OCR/ECAR ratio.

## Conclusion

**Figure 7:**
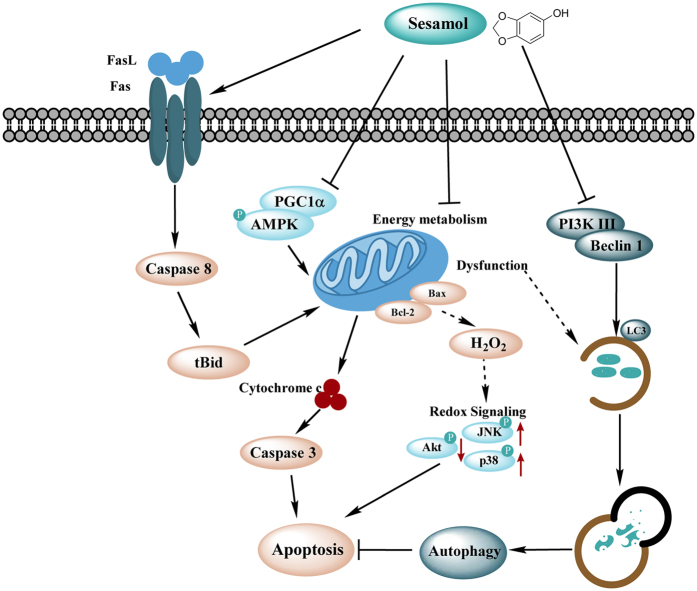
Illustration of the mechanism of sesamol-induced apoptosis in HepG2 cells.

In current study, sesamol has been shown to induce apoptosis by impairing mitochondria in hepatocellular carcinoma *in vitro* and *in vivo*. As shown in Fig. 7, our results indicated that sesamol induced loss of mitochondrial membrane potential and defected mitochondrial energy metabolism, followed by excess H_2_O_2_ production and redox-sensitive signaling disturbance. Sesamol arrested cell cycle at S phase and activated both the intrinsic and extrinsic pathways of apoptosis. Moreover, sesamol suppressed mitochondrial biogenesis and mitophagy leading to the accumulation of more defective mitochondria to induce cell death. Therefore, our study suggests mitochondrion play a central role in sesamol-induced hepatocellular carcinoma apoptosis.

## Materials and Methods

### Cell culture and Sesamol treatment

HepG2 cells were provided by Kunming Institute of Zoology, Chinese Academy of Sciences (Kunming, China) and cultured in Dulbecco’s minimal essential medium (DMEM) (Gibco Co., USA) supplemented with 10% FBS, 100 IU/mL penicillin, and 100 μg/mL streptomycin at 37 °C in a humidified atmosphere with 5% CO_2_. For the sesamol treatment, sesamol was dissolved in dimethyl sulfoxide (DMSO) to a stock solution of 1 M and further diluted to different concentrations with FBS-free culture medium. For rapamycin treatment, HepG2 cells were pre-treated with/without 100 nM rapamycin for 30 min, and then treated with 1 mM sesamol.

### Reagents and antibodies

Oligomycin, rotenone, and carbonyl cyanide 4-(trifluoromethoxy) phenylhydrazone (FCCP) were purchased from MP Medicals (Solon, OH, USA). Sesamol, rapamycin, and protease/phosphatase inhibitor cocktail were purchased from Sigma Chemical Co. (St Louis, MO, USA). The primary antibodies against Bcl-2, Bax, FasL, ND1, cytochrome *c*, PGC1α, β-actin, and HRP-labeled secondary antibodies were purchased from Santa Cruz Biotechnology (Dallas, Texas, USA). Fas, cleaved caspase-3, Bid, procaspase-8, PARP, Akt, p-Akt, JNK, p-JNK, p38, p-p38, PI3K III, Beclin-1, Ki67, and LC3 I/II antibodies were purchased from Cell Signaling (Danvers, MA, USA).

### Colony formation assay

Colony formation assays were performed as previously described with minor modification^57^. Cells were plated in 6-well plates at a concentration of 1.0 × 10^3^ cells/well and were allowed to grow for overnight. Then, cells were incubated in the presence or absence of various concentrations (0, 0.1, 0.25, 0.5, 1, 2 mM) of sesamol for 24 h. The sesamol-containing medium was then removed, and the cells were washed in PBS and incubated for an additional 7 d in complete medium (10% FBS) to form colonies. Subsequently, cells were washed twice with PBS, treated with crystal violet for 10 min, washed three times with PBS, and then photographed with a digital camera.

### MTT assay

The cytotoxicity of sesamol on HepG2 cells and BRL-3A cells was examined by using MTT assay as previous study described^58^. Briefly, cells in exponential growth were seeded into 96-well plates at a density of 1 × 10^5^ cells/mL. The cells were then treated with sesamol at various doses (0, 0.01, 0.05, 0.1, 0.2, 0.5 or 1 mM). After 24 h, the supernatant was discarded and 100 μL FBS free medium was added. 100 μL of MTT was added in each well, and cells were further incubated for 4 h in the dark. After incubation, the medium was discarded, 100 μL of DMSO was added, and the optical density of each well was measured at 570 nm using a Bio-Rad Model 680 microplate reader (Bio-Rad Laboratories, Hercules, CA, USA).

### Flow cytometry assay

For apoptosis detection, cells were collected and resuspended at a concentration of 1 × 10^6^ cells/mL in binding buffer (10 mM HEPES/NaOH (pH 7.4), 140 mM NaCl, 2.5 mM CaCl_2_) and incubated with FITC conjugated Annexin V and propidium iodide (PI) (Life Technologies, Grand Island, NY, USA) for 15 min at room temperature.

For cell cycle studies, HepG2 cells were treated with PI for 15 min after 24 h incubation with varying concentrations of sesamol. For the autophagy marker LC3 detection, HepG2 cells were transfected with Premo™ Autophagy Sensor combines the selectivity of a LC3B-GFP (Thermo, USA, Catalog number: P36235) for 16 h and then treated with 1 mM sesamol for 24 h. GFP-LC3, cell cycle and apoptosis was then detected using the flow cytometer CyFlow Cube (Partec Inc., German). The data was analyzed by FCS express 5 plus (*De Novo* Software).

### Analysis of MMP (Δψ)

Mitochondrial membrane potential was determined using the mitochondrion-specific lipophilic cationic fluorescence dye JC-1 (Beyotime Institute of Biotechnology, Haimen, Jiangsu, China) as described in previous research^58^. Cells were planted into 96-well plates (7.0 × 10^4^ cells/well) and incubated with different concentrations of sesamol for 4 or 24 h. Cells were then treated with 5 μg/mL JC-1 for 0.5 h at 37 °C and were washed twice with PBS. Fluorescence intensity was measured using a multimode microplate reader (Molecular Devices Co., Sunnyvale, CA, USA) at 485 nm excitation, and 585 and 538 nm emission, respectively. The values were expressed as the OD585/OD538 ratio.

### H_2_O_2_ measurements

H_*2*_O_*2*_ generation from HepG2 cells was determined by the Amplex Red Hydrogen Peroxide/Peroxidase Assay kit (Invitrogen) following the manufacturer’s instructions.

### Mitophagy detection

HepG2 cells were subcultured in a cell culture chamber (LAB-Tek^TM^, Thermo Scientific, USA) pre-coated with collagen and incubated overnight at 37 °C with 5% (*v/v*) CO_2_. Cells were transfected with GFP-LC3 virus for 16 h, then pre-treated with/without 100 nM rapamycin for 30 min, and then treated with 1 mM sesamol for 24 h. The green channel of the images demonstrated GFP-LC3 expression. Mitochondria were labelled with MitoTracker-red kit (Thermo, USA, Catalog number: M7512). Cells were observed using a fluorescence microscope (Olympus Optical Co., Ltd., Tokyo, Japan). Red channel demonstrated mitochondria. In the overlay channel, the mitophagy was shown as orange.

### Observation of MDC and AO/EB staining

MDC staining was introduced as a specific autophagosome marker to analyze the autophagic process. After treatment, cells were washed with PBS twice and incubated with 0.05 mM MDC in RPMI1640 for 30 min. After washing twice with PBS, the cellular fluorescence was visualized by using fluorescence microscopy (Olympus Optical Co., Ltd., Tokyo, Japan). AO/EB staining was applied to investigate whether cells underwent cell death via apoptosis, since AO can pass through the cell membrane, but EB cannot. A monolayer of HepG2 cells was incubated with sesamol (1 mM) with or without rapamycin (100 nM) pre-treatment for 24 h. Then each cell culture was stained with AO/EB solution (100 mg/mL AO in PBS, 100 mg/mL EB in PBS). Samples were visualized by using fluorescence microscopy mentioned above.

### Western blotting

Cell lysate was solubilized in SDS sample buffer and the cytosolic components and mitochondria were isolated as described in the supplementary materials. Samples were separated by Laemmli SDS/PAGE, and transferred onto PVDF membranes. Using appropriate antibodies, the immunoreactive bands were visualized with an enhanced chemiluminescence reagent.

### Metabolic flux analysis

Metabolic flux analysis was performed as described in ref. 59: Briefly, HepG2 cells were cultured on Seahorse XF-24 (Seahorse BioSciences, Billerica, MA, USA) plates at a density of 7.0 × 10^4^ cells per well. Cells were treated with control vehicle, sesamol (1 mM), rapamycin (100 nM), or sesamol (1 mM) + rapamycin (100 nM). Cells were pre-treated with rapamycin for 30 min and the assays were conducted 24 h post-treatment. On the day of metabolic flux analysis, the media was changed to unbuffered DMEM medium and treated at 37 °C in a non-CO_2_ incubator for 1 h. All medium and injection reagents were adjusted to pH 7.4 on the day of assay. Using the Seahorse XF-24 (Seahorse BioSciences) metabolic analyzer, three baseline measurements of OCR and ECAR were sampled prior to sequential injection of mitochondrial inhibitors. Three metabolic determinations were sampled following addition of each mitochondrial inhibitor prior to injection of the subsequent inhibitors. The mitochondrial inhibitors used were oligomycin (4 μM), FCCP (0.2 μM), and rotenone (1 μM). OCR and ECAR were automatically calculated and recorded by the Seahorse X-24 analysis software. After the assays, protein levels were determined by BCA assay for each well to confirm equal cell density per well.

### Xenograft model

Five-week-old male nude mice (Balb/c nu/nu) were purchased from Model Animal Research Center of Nanjing University (Nanjing, China). All of the experimental procedures followed by the Guide for the Care and Use of Laboratory Animals: Eighth Edition, ISBN-10: 0-309-15396-4, and the animal protocol was approved by the animal ethics committee of Xi’an Jiaotong University. All surgery was performed under anesthesia and all efforts were made to minimize suffering. The mice were implanted subcutaneously in their right flanks with tumor cells suspended in PBS. HepG2 cells (1 × 10^7^) in 0.2 mL of PBS were injected subcutaneously between the scapulae of each nude mouse. After transplantation, the tumor sizes were measured using calipers, and the tumor volumes were estimated^60^. Once the tumors had reached a mean size of 100 mm^3^ (day 10), the mice were randomly divided into three groups (5 animals per group). The treatment group received sesamol treatment via i.p. injection every other day from day 10 thru day 44 (100 mg/kg or 200 mg/kg per day), and the control group received the same volume of saline (vehicle). The bodyweight and tumor volume of each mouse was assessed every three days using caliper measurements. On day 45, the mice were sacrificed and the tumor tissues was either cut into several portions for use in western blot analyses or stored at −80 °C.

### Immunohistochemical staining of tumor samples

Tumor samples were obtained and immediately fixed with paraformaldehyde. After normal processing, the samples were embedded in paraffin and sliced. The Ki67, cleaved-caspase 3, LC3 antibody was performed in this study. The staining was imaged by microscopy.

### Statistical analysis

Data are reported as means ± SD or SEM of at least three independent experiments. Significant differences between mean values were determined by Student’s t-test. Means were considered to be statistically distinct if p < 0.05.

## Additional Information

**How to cite this article:** Liu, Z. *et al*. Sesamol Induces Human Hepatocellular Carcinoma Cells Apoptosis by Impairing Mitochondrial Function and Suppressing Autophagy. *Sci. Rep.* **7**, 45728; doi: 10.1038/srep45728 (2017).

**Publisher's note:** Springer Nature remains neutral with regard to jurisdictional claims in published maps and institutional affiliations.

## Supplementary Material

Supplementary Figure S1 and S2

## Figures and Tables

**Table 1:** Effects of sesamol treatment on the bioenergetic parameters in HepG2 cells.

	Oxygen consumption rates (pmol/min)			
	Control	sesamol	rapamycin	sesamol + rapamycin
Basal respiration	175 ± 4	152 ± 12^*^	193 ± 5^**^	162 ± 10
OXPHOS-induced respiration	148 ± 4	133 ± 12	162 ± 5^**^	136 ± 10
H^+^ leak-induced respiration	26 ± 10	19 ± 5	30 ± 9	26 ± 6
Maximal respiratory capacity	365 ± 10	317 ± 11^**^	410 ± 3^**^	378 ± 33^##^
Nonmitochondrial respiration	75 ± 9	83 ± 12	85 ± 2	87 ± 6
Reserve capacity	190 ± 10	165 ± 11	217 ± 3	216 ± 33

Assay conditions as described in the Methods and Materials. *p ≤ 0.05, **p ≤ 0.01 *versus* control group. ^##^p ≤ 0.01 *versus* Sesamol group.
