# Supplementary material for: Sesamol Induces Human Hepatocellular Carcinoma Cells Apoptosis by Impairing Mitochondrial Function and Suppressing Autophagy
Source: Sci Rep. 2017 Apr 4;7:45728. doi: 10.1038/srep45728 (PMC5379556; doi:10.1038/srep45728)
Supplement: Supplementary Figure S1 and S2 [file srep45728-s1.pdf]

Sesamol Induces Human Hepatocellular Carcinoma Cells Apoptosis by Impairing Mitochondrial Function and Suppressing Autophagy

Zhigang Liu[1], Bo Ren[1], Yihui Wang[2], Chen Zou[3], Qinglian Qiao[1], Zhijun Diao[1], Yashi Mi[1], Di Zhu[1], Xuebo Liu[1]*

1 Laboratory of Functional Chemistry and Nutrition of Food, College of Food Science and Engineering, Northwest A&F University, Yangling, Shaanxi 712100, China

2 School of Medicine, Tulane University, New Orleans, LA 70112, USA

3 School of Agriculture and Biology, Shanghai Jiao Tong University, Shanghai 200240, China

* Correspondence to: Dr. Xuebo Liu, College of Food Science and Engineering, Northwest A&F University, 28. Xi-nong Road, Yangling 712100, China.

Tel: +86-029-87092325; Fax: +86-029-87092325; E-mail: xueboliu@aliyun.com

Materials and methods for supplemental data

DCF staining assay

The cell-permeant H_2DCFDA (2',7'-dichlorodihydrofluorescein diacetate) (purchased from Beyotime Institute of Biotechnology, Haimen, Jiangsu, China) was used as an expression of the cell's redox status; The BRL-3A cells in exponential growth were seeded into 96-well plates at a density of 1×10^5 cells/mL overnight. The cells were then treated with sesamol at various doses (0, 0.25, 0.5, 1 mM). After 24 h, each well was incubated for 30 min with 10 μL of the dye and fluorescence intensity fluorescence intensity was measured using a multimode microplate reader (Molecular Devices Co., Sunnyvale, CA, USA) at 488 nm excitation and 525 nm emission.

Cytosolic components and mitochondria isolation

After various concentration of sesamol treatment for 24 h, fresh cells were collected to isolate cytosolic components and mitochondria using Mitochondrial isolation kit (purchased from Beyotime Institute of Biotechnology, Haimen, Jiangsu, China). Briefly, the cells were homogenized (10-30 strokes) on ice followed by 600 g centrifuge for 10 min at 4 °C. Then carefully transfered the supernatant to a new fresh tube and centrifuge at 11,000 g for 10 min at 4 °C. The supernatant was the cytosolic components and the pellet was the mitochondria. The cytochrome c was detected in the cytosol using western blotting assay.

Glucose consumption

HepG2 cells in exponential growth were seeded into 96-well plates at a density of 1×10^5 cells/mL overnight. The cells were then treated with sesamol at various doses (0, 0.25, 0.5, 1 mM). After 24 h, the medium supernatant was collected respectively. In accordance with instructions of the glucose diagnostic kit (E1010, purchased from Applygen, Beijing, China), the mediums were measured at 570 nm with enzyme standard instrument. According to the optical density of each well and the standard curve of glucose, the glucose content in the medium was calculated. The glucose content of the experimental groups and the original DMEM mediums were measured. Glucose consumption was the difference value between the original DMEM mediums and the medium supernatant of the experimental group medium.

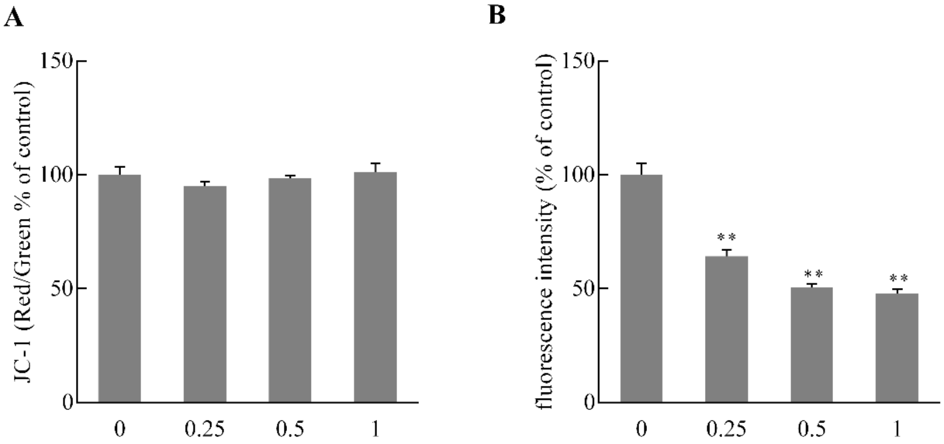

Supplementary Figure S1 *Effects of sesamol on mitochondrial membrane potential and redox status of BRL-3A*

BRL-3A cells were treated with sesamol at the indicated concentrations for 24 h. After treatment, (A) the cells were detected by a multimode reader after staining with 5 μg/mL JC-1; the bar graph is the fluorescence intensity which was measured using a multimode microplate reader at 485 nm excitation, 585 nm (red/orange for normal MMP) and 538 nm (green for loss of MMP) emission, respectively. (200×, magnification). (B) Cellular redox status was examined by DCF staining as Materials and methods section described. Data presented as mean ± SD, n=8 wells per group, $^{*}p<0.05$, $^{**}p<0.01$ versus control group.

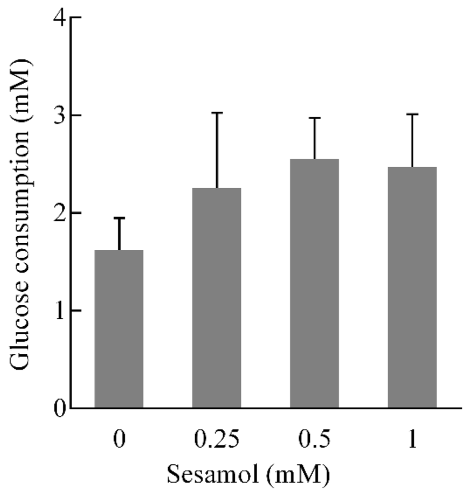

Supplementary Figure S2 *Effects of sesamol on glucose consumption of HepG2 cells*

HepG2 cells were treated with sesamol at the indicated concentrations for 24 h. After treatment, the glucose content of the experimental groups and the original DMEM mediums were measured by glucose diagnostic kit. Glucose consumption was the difference value between the original DMEM mediums and the medium supernatant of the experimental group medium. Data presented as mean ± SD, n≥6 wells per group.
